# Understanding Monoclonal B Cell Lymphocytosis: An Interplay of Genetic and Microenvironmental Factors

**DOI:** 10.3389/fonc.2021.769612

**Published:** 2021-11-11

**Authors:** Chrysi Galigalidou, Laura Zaragoza-Infante, Anastasia Iatrou, Anastasia Chatzidimitriou, Kostas Stamatopoulos, Andreas Agathangelidis

**Affiliations:** ^1^ Institute of Applied Biosciences (INAB), Centre for Research and Technology Hellas (CERTH), Thessaloniki, Greece; ^2^ Department of Molecular Biology and Genetics, Democritus University of Thrace, Alexandroupolis, Greece; ^3^ Hematology Department, University General Hospital of Thessaloniki AHEPA, Thessaloniki, Greece; ^4^ Department of Molecular Medicine and Surgery, Karolinska Institutet, Stockholm, Sweden; ^5^ Department of Biology, School of Science, National and Kapodistrian University of Athens, Athens, Greece

**Keywords:** monoclonal B cell lymphocytosis (MBL), chronic Lymphocutic Leukemia (CLL), genetics, immunogenetics, tumor microenvironment, ontogenesis, B cell receptor, immunoglobulin

## Abstract

The term monoclonal B-cell lymphocytosis (MBL) describes the presence of a clonal B cell population with a count of less than 5 × 10^9^/L and no symptoms or signs of disease. Based on the B cell count, MBL is further classified into 2 distinct subtypes: ‘low-count’ and ‘high-count’ MBL. High-count MBL shares a series of biological and clinical features with chronic lymphocytic leukemia (CLL), at least of the indolent type, and evolves to CLL requiring treatment at a rate of 1-2% per year, whereas ‘low-count’ MBL seems to be distinct, likely representing an immunological rather than a pre-malignant condition. That notwithstanding, both subtypes of MBL can carry ‘CLL-specific’ genomic aberrations such as cytogenetic abnormalities and gene mutations, yet to a much lesser extent compared to CLL. These findings suggest that such aberrations are mostly relevant for disease progression rather than disease onset, indirectly pointing to microenvironmental drive as a key contributor to the emergence of MBL. Understanding microenvironmental interactions is therefore anticipated to elucidate MBL ontogeny and, most importantly, the relationship between MBL and CLL.

## Introduction

### Epidemiology

Monoclonal B cell lymphocytosis (MBL) is an asymptomatic hematological condition characterized by the presence of clonal B cell expansion(s) in otherwise healthy individuals. The immunophenotype of the B cell clones in approximately 75% of all MBL cases is similar to that observed in chronic lymphocytic leukemia (CLL): CD5^+^, CD19^+^, CD20^dim^, CD23^+^ and low levels (dim) of surface immunoglobulin (sIg^dim^) that in most cases is IgM with or without IgD. Other immunophenotypic characteristics include the absence of FMC7 and the weak or no expression of CD79b and CD22. Based on this constellation of features, the most common subtype of MBL is defined as ‘CLL-like’. Another immunophenotype occasionally observed in MBL is characterized by strong expression of CD20 and sIg along with absence of CD23 (CD5^+^, CD19^+^, CD20^high^, CD23^+^, sIg^high^); this MBL subtype is described as ‘atypical’ MBL. The last, most infrequent, MBL subtype is ‘CD5-negative’ MBL, in which the complete immunophenotypic characterization is CD5^neg^, CD19^+^, CD20^+^ with moderate to high sIg expression, with the ratio of κ: λ sIg being strongly skewed (either more than 3:1 or less than 1:3) (1–4).

### Immunophenotypic Subtypes of MBL

Significant differences exist between the three MBL immunophenotypic subtypes regarding the expression of several cell surface markers, such as CD23, CD79b, and FMC7. Moreover, several established CLL risk factors are also differentially expressed between the immunophenotypic subtypes of MBL; for instance, markers associated with aggressive CLL, such as CD38, ZAP70, and CD49d are highly expressed in the majority of ‘atypical’ MBL cases yet are rarely expressed in ‘CLL-like’ MBL (1, 5). Moreover, the B cell memory marker CD27 shows higher expression in the ‘CLL-like’ type of MBL compared to ‘atypical’ and ‘CD5-negative’ MBL, while, in contrast, CD22, CD79a, CD79b, and CD1c (BDCA-1) are mainly expressed in the ‘non CLL-like’ types. Furthermore, FMC7 and CD43 are also characterized by differential expression among the three immunophenotypic types of MBL; more specifically, FMC7 exhibits low expression levels in ‘CLL-like’ MBL, intermediate levels in atypical, and high levels in the ‘CD5-negative’ type of MBL, while the exact opposite pattern is evident for CD43 expression (6). Differences are also observed regarding the expression of sIg, which is significantly lower in ‘CLL-like’ MBL compared to either the ‘atypical’ or the ‘CD5-negative’ types (1). Finally, expression of the *ROR1* gene was statistically different among the three immunophenotypic subtypes of MBL being significantly higher in ‘CLL-like’ MBL versus the others (1).

### MBL Risk Factors

Moving from the immunophenotype, the diagnostic criteria for distinguishing between MBL from CLL are primarily based on the number of circulating monoclonal B cells. Two distinct MBL subtypes are recognized: high-count MBL (HC-MBL), or ‘clinical’ MBL, with 0.5-4.99× 10^9^ clonal B cells/L and low-count MBL (LC-MBL), or ‘general’ population MBL, characterized by <0.5 × 10^9^ clonal B cells/L (4). It should be noted that most individuals diagnosed with MBL display a normal absolute B cell count, with the clonal B cell population accounting for less than 10% of the total number of B cells (3).

The incidence of MBL gradually increases with advancing age; in fact, MBL is very rarely detected in individuals under 40 years old, whereas it is present in more than 20% of individuals older than 70 years old and can reach even 75% among patients aged more than 90 years (3). Age is not the only factor strongly associated with MBL, as a familial history of hematological or solid malignancies has also been reported; in fact, 17% of first-degree relatives with no personal history of lymphoproliferative disorders from families with at least two cases of CLL were found to have MBL, in some cases at a young age (less than 40 years old) (7). Gender is another factor connected to the prevalence of MBL as, similar to CLL, males have a significantly higher risk for developing MBL compared to females (8).

Exposure to infections and immunodeficiency may also predispose to MBL development, at least in certain instances, especially when combined with advanced age and male gender. A characteristic example concerns the high frequency of MBL among patients with hepatitis C virus (HCV) infection (9). On the other hand, MBL was found to be significantly less common among individuals vaccinated against pneumococcal or influenza infections (3).

### Risk of Progression to CLL

Unmutated somatic hypermutation (SHM) status of the rearranged immunoglobulin heavy variable (IGHV) gene and/or ‘CLL-related’ cytogenetic aberrations related to aggressive disease represent risk factors of progression from MBL to CLL and shorter overall survival (OS) (10). Furthermore, over 50% of HC-MBL cases carry at least one gene mutation in a CLL putative driver, evident up to 41 months before the progression to CLL. Thus, genomic characterization can be used to identify those MBL cases who will progress to CLL requiring treatment; this was more pronounced in cases with subclonal expansion of driver mutations (11, 12).

Along these lines, the genetic distance between LC-MBL and CLL is clearly greater than that of HC-MBL (13) and, thus, the question whether LC-MBL represents a very early stage in the natural history of CLL, remains elusive.

## HC-MBL progressing to CLL

Monoclonal B cell populations can be detected long before CLL diagnosis, even up to 6.4 years (13). However, the majority of ‘CLL-like’ HC-MBL cases remain stable overtime; progression to CLL has been estimated at a rate of 1-4% per year (1). In more detail, a longitudinal analysis in 185 individuals with ‘CLL-like’ HC-MBL revealed a progressive lymphocytosis in 28% of the cases with a median follow-up of 6.7 years. However, only 7% of the cases progressed to CLL requiring treatment with the overall rate of progression to CLL being 1.1% per year (14). In a retrospective study by the Mayo Clinic, 302 ‘CLL-like’ HC-MBL cases were monitored for a median of 18 months with ~1.4% of cases per year requiring treatment due to disease progression. This study proposed a B cell count threshold of 10 x 10^9^/L for discriminating between two categories of ‘CLL-like’ HC-MBL cases with higher and lower probability of treatment. This proposal was subsequently tested and validated in the GIMEMA database (15, 16). Finally, in an Italian study, 123 ‘CLL-like’ HC-MBL cases were monitored for a median of 43 months with 4% per year requiring treatment in the first 7 years (17). Irrespective of progression to CLL, periodic hematological follow-up was recommended after the detection of HC-MBL, since this group has shorter overall survival compared to the general population (18).

Many of the biological characteristics of HC-MBL are similar to CLL Rai stage 0 (19). Nowadays, the strongest predictive marker for MBL progressing to CLL is the B cell count. This has emerged after the establishment of a B cell count-based system transition for discriminating MBL from CLL. This has led to a sizeable fraction of patients, considered as Rai stage 0 CLL, ending up classified as HC-MBL (20, 21). Relevant findings suggested that the B cell count appears to be a better predictor of TFS and OS when a defined lymphocyte threshold is applied (10). In a relevant study, the authors proposed a distinction of 2 ‘CLL‐like’ cell count thresholds for the identification of a very low risk group (<1.2x10^9^/L) and a high risk group (>3.7x10^9^/L) within HC-MBL (10, 17).

Subsequent studies appraised the impact of the size of the MBL clone on several clinical metrics, such as progression-free-survival (PFS), TFS and OS. In regard to the former, it is rather difficult to draw robust conclusions, given that a slight increase in cell numbers in HC-MBL cases with clone sizes near the mathematical cutoff of 5.0x10^9^/L could be classified as disease progression. On the other hand, the assessment of TFS through standard NCI/iwCLL criteria may be more informative (22).

In CLL, the study of prognostic markers for time-to-first-treatment (TTFT) and OS has been of great interest, especially towards the development of a multifactorial risk score. In this context, 28 individual prognostic variables were assessed in a series of 969 individuals with CLL Rai 0 or HC-MBL leading to the development of the CLL international Prognostic Index (CLL-IPI). Implementation of the CLL-IPI score led to the identification of 4 risk groups (low, intermediate, high, and very-high risk) with different 5-year OS (93%, 79%, 63%, and 23%, respectively). Multivariate analysis of absolute B-cell count with individual factors of the CLL-IPI showed that five parameters, namely age, Rai stage, serum beta-2 microglobulin, unmutated IGHV genes, and del(17p) or *TP53* mutation were associated with shorter TTFT and OS. Overall, the CLL-IPI risk score, despite some limitations, is able to predict TTFT and OS in previously untreated CLL patients whose only disease symptom is the presence of a circulating clonal B cell population (23). Despite these advances, the relevance of CLL-IPI for the evolution of HC-MBL to CLL remains to be fully elucidated.

Several markers have been studied at MBL diagnosis for their capacity to predict progression to CLL, including B cell receptor immunoglobulin (BcR IG) stereotypy, ZAP70 expression, Hb, platelet count and LDH, however none of them was clearly associated with progression to CLL (p≥0.05 in all instances). In contrast, the SHM status of the clonotypic IGHV gene, high expression of CD38 (≥30%), high expression of CD49d (≥30%) and chromosomal abnormalities were found to be significant on univariate analysis regarding prediction of TTFT. However, on multivariate analysis trisomy 12 and del (16) (p13) were the sole independent predictors of treatment-free survival (TFS) in MBL after adjusting for IGHV SHM status and CD38 expression (5, 14).

## Persistence of LC-MBL over time

HC-MBL represents the vast majority of MBL cases (approximately 85%) identified in the context of clinical practice following the investigation of lymphocytosis, with a median ‘CLL-like’ B cell count above 1.9x10^9^/L. On the contrary, around 85% of MBL cases detected in population screening studies have a ‘CLL-like’ B cell count below 0.5x10^9^/L, with 40% of them bearing fewer than 0.05x10^9^ clonal B cells/L (24). Previous findings support a bimodal distribution with a lower peak below 0.05x10^9^ clonal B cells/L, very few cases in the range of 0.05-0.5 x10^9^ clonal B cells/L, and another peak accounting for cases with a clonal B cell count ranging between 0.5-5x10^9^ clonal B cells/L. A possible explanation could be a bias towards the selection of cases with high lymphocyte counts against those with mild or borderline lymphocytosis in the hematology clinical routine (22). An alternative biological explanation is offered by the distinct immunogenetic profiles of HC-MBL and LC-MBL, whereby pronounced clonal expansions are seen only when cells with ‘CLL-like’ phenotype express BcR IG with certain distinctive features (19). Longitudinal studies support the latter explanation, since most of the LC-MBL cases persisted over large periods of time (up to 34 months), without any progression to clinically overt disease. In fact, LC-MBL seems to be stable over time, a characteristic that is more pronounced in ‘CLL-like’ LC-MBL (90%) compared to other immunophenotypic variants, such as atypical (44.4%) and CD5-negative MBL (66.7%) (2).

Even though progression to HC-MBL is very rare, LC-MBL cases may often display a small increase in the size of the B cell clone. Studies focusing on clonal dynamics revealed a correlation between clonal size and clonality; in specific, the majority of monoclonal cases increased in size over time, while a much smaller number of biclonal and multiclonal cases behaved accordingly (25). Mathematical modeling of these results led to the conclusion that, in most cases, the estimated time for the progression of LC-MBL to CLL far exceeds a normal life expectancy. However, assessment of the clinical impact of LC-MBL, if any, through the comparison against age- and sex-matched non-MBL subjects showed that the OS observed for individuals with LC-MBL was significantly shorter than that of the control group. Moreover, individuals with LC-MBL showed a significantly shortened survival compared to that of age-matched individuals of the general population from the same geographical region. Infections, cancer and cardiovascular diseases were the main causes of mortality among individuals with LC-MBL. On the other hand, non-infectious respiratory tract diseases or genitourinary diseases, diabetes, dementia or other nervous system disorders accounted for almost 30% of deaths in the age- and sex-matched general population cohort. In the entire cohort, advanced age, co-existing cardiovascular diseases, solid tumors and, to a lesser extent, the presence of LC-MBL clones, were independently associated with a shorter OS (25).

## ‘CLL-specific’ cytogenetic aberrations are detected in MBL

The cytogenetic profile of HC-MBL is highly similar to that of CLL, especially of the indolent type (Rai 0 stage), in that del(13q14) was found to be the most frequent aberration followed by trisomy 12 (1). On the other hand, compared to Rai 0 CLL, HC-MBL displayed a lower prevalence of cytogenetic aberrations related to aggressive disease, such as del(11q) and del(17p), that were often acquired as secondary abnormalities (17).

**Figure 1:**
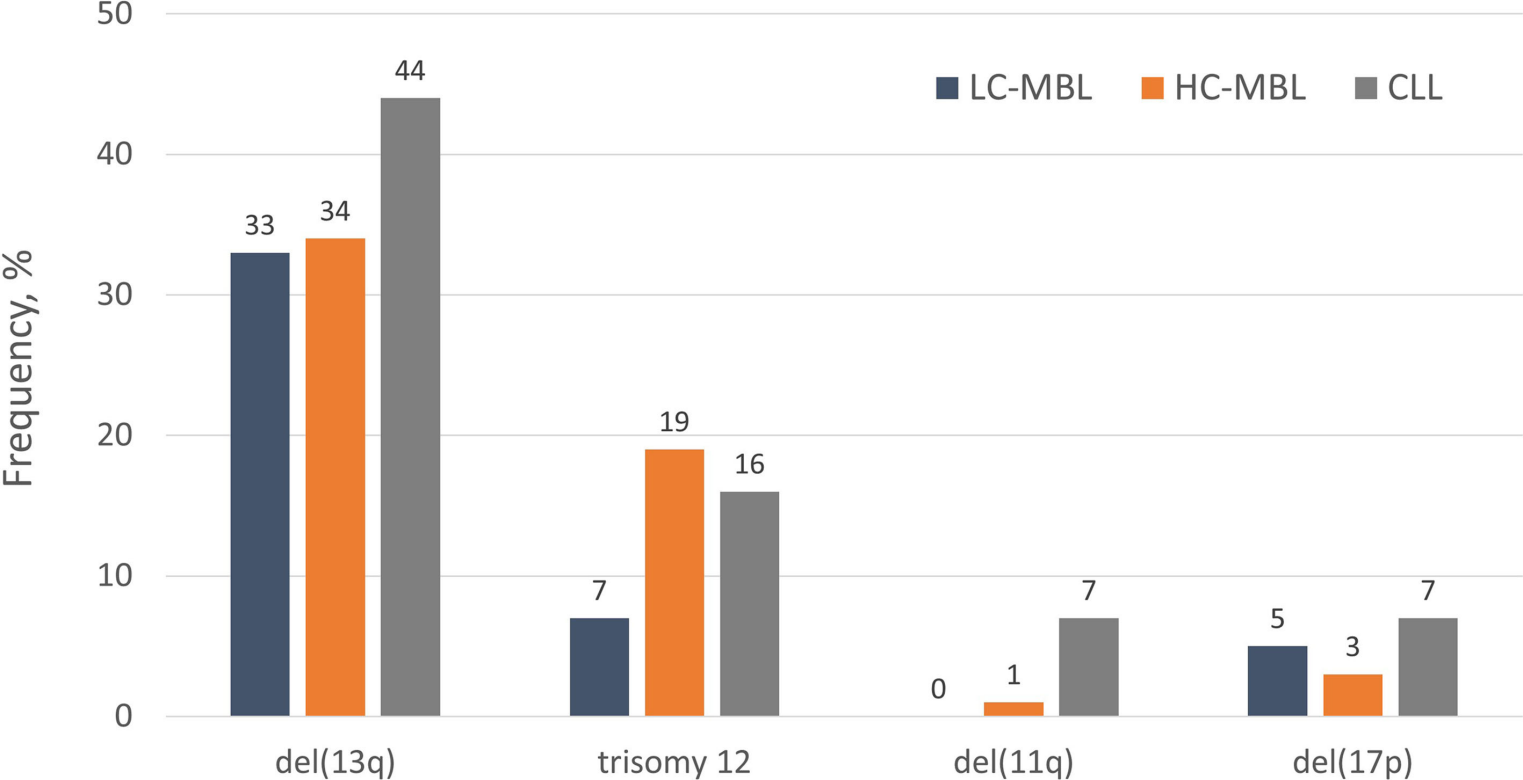
The distribution of “CLL-specific” cytogenetic aberrations in LC-MBL, HC-MBL and indolent CLL. Data was extracted from the studies by Rossi et al (17), Fazi et al. (2) and Henriques et al. (26).

In contrast, LC-MBL showed a significantly lower frequency of ‘CLL-specific’ cytogenetic aberrations compared to HC-MBL and CLL (26). At the individual aberration level, a tendency was observed towards a greater frequency of del(13q) and trisomy 12 from LC-MBL to HC-MBL and CLL, yet the individual frequencies were not significantly different between the three. On these grounds, one could speculate that these lesions occur rather early during the development of MBL and may be associated with the acquisition of the ‘CLL-like’ immunophenotypic profile rather than overt disease (26). A much lower prevalence of del(11q) and del(17p) was observed in LC-MBL; even so, such cases were detected, with the aberration being present in the majority of clonal B cells (2). This finding indicates that the presence of del(17p) per se does not axiomatically correlate with aggressive disease, as also reported for a small group of patients with CLL carrying this abnormality yet remaining stable for prolonged periods, especially when expressing mutated IGHV genes (27, 28). The distribution of the most common “CLL-related” cytogenetic aberrations among LC-MBL, HC-MBL and CLL is depicted in **Figure 1**. Of note, although LC-MBL appears to display a relatively simple cytogenetic profile, longitudinal analysis disclosed a significant increase in the overall frequency of cytogenetic aberrations after seven years of follow-up (29% at baseline versus 62% at follow-up): importantly, all cytogenetic aberrations observed at baseline also persisted at follow-up (25).

Overall, these findings support that the progression from LC-MBL to HC-MBL and CLL is accompanied by the progressive acquisition of recurrent cytogenetic aberrations, each one of them enriched at specific stages along the natural history of the disease. In more detail, del(13q) and trisomy 12 occur rather early, at the LC-MBL stage, whereas del(17p) and del(11q) seem to emerge later, as secondary events at either the HC-MBL or the CLL stages. In line with this, the study of telomere length (TL) in both subtypes of MBL, indolent CLL and healthy subjects revealed the presence of significantly shorter telomeres already at the level of HC-MBL, suggesting that it may be part of the initial events in the process of CLL pathogenesis (26, 29).

Very few data is available for the profile of cytogenetic aberrations in ‘atypical’ and ‘CD5-negative’ subtypes of MBL; having said that, cases with del(13q), trisomy 12 and even del(17p) have been identified (1).

## Recurrent gene mutations in MBL

*TP53* aberrations, including mutations, have an established role in shaping the outcome of CLL and, for this reason, their assessment is considered mandatory before treatment initiation (30). In recent years, the advent of NGS technologies led to the identification of additional gene mutations with putative clinical relevance in CLL. In particular, recurrent mutations in the *NOTCH1*, *SF3B1*, and *BIRC3* genes were associated with distinct outcomes and were utilized in risk stratification schemes in the pivotal studies (31–33). Subsequent studies by several groups, including ours, have identified mutations in additional genes, e.g. *RPS15*, *NFKBIE* and *EGR2*, revealing a much more complicated genetic landscape for CLL (34–36). To complicate things even more, the detailed study of the clonal architecture in CLL in comparison to treatment revealed that the presence of mutations in putative CLL driver genes can adversely impact the clinical outcome of the disease, even when present in minor subclones, at least for certain mutations (37).

**Figure 2:**
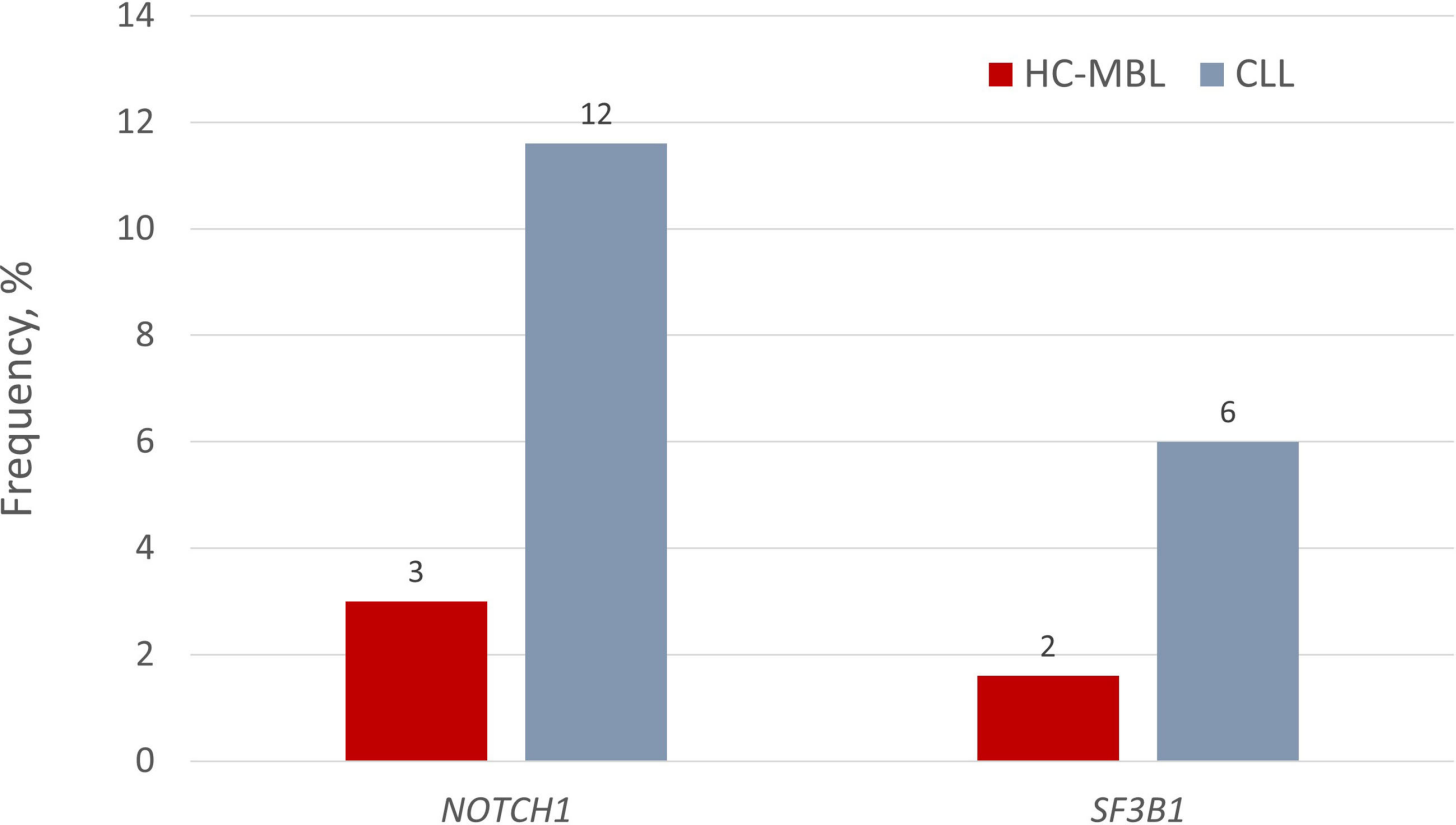
Incidence of *NOTCH1* and *SF3B1* gene mutations in HC-MBL and indolent CLL. Respective values for LC-MBL were 0% in both cases. Data regarding the presence of gene mutations in LC-MBL was extracted from Agathangelidis et al. (40), data regarding *NOTCH1* mutations in HC-MBL and CLL from the study by Rasi et al. (38), and finally, the frequency of *SF3B1* mutations in HC-MBL and CLL from the studies by Greco et al. (39) and Rossi et al. (33), respectively.

Some of the same recurrent gene mutations have been also found in HC-MBL. That said, at the individual gene level, *NOTCH1* mutations exhibited a significantly lower prevalence in HC-MBL compared to CLL (3.2% versus 11.6%, respectively) (P=0.050) (38); moreover, *SF3B1* mutations were even more scarce in HC-MBL (1.5%) versus CLL (~10%) (39). Interestingly, however, when larger cohorts were analyzed, the incidence of *NOTCH1* and *SF3B1* mutations was not significantly different between HC-MBL and Rai 0-CLL (*NOTCH1*: 8.2% *vs.* 13.1%; *SF3B1*: 4.7% *vs.* 3.8%) (**Figure 2**) (21). This finding supports the claim that MBL and early stage CLL are closely similar, differing only in clonal size, with MBL simply requiring more time to expand. This claim is further corroborated by the fact that low variant allele frequencies (VAF) were observed in almost all MBL cases but only in about half of CLL cases (41).

At the clinical level, both CLL and MBL cases bearing a *NOTCH1* mutation had a shorter progression free survival (PFS) compared to wildtype ones (41). Thus, arguably, gene mutations could be used as biomarkers for identifying those MBL cases that would eventually progress to CLL requiring treatment. Indeed, in a longitudinal analysis, 60% of MBL cases that progressed to require treatment exhibited a subclonal expansion bearing a driver gene mutation, compared to only 14% of untreated cases (11). On the other hand, a longitudinal analysis of eight MBL cases by whole exome sequencing (WES) at two time points of 65 months apart, revealed that the four cases who developed detectable lymphadenopathy by physical exam yet without need for treatment carried mutations in putative CLL driver genes, including *ATM*, *DDX3X*, *EGR2*, *FBXW7*, *SAMHD1* and *SF3B1* (42). Targeted re-sequencing not only validated the WES results, but also led to the identification of additional mutations in the *BIRC3, POT1* and *NOTCH1* genes. Interestingly, a damaging mutation affecting *PRDM1* was identified in a fifth case, which is frequently inactivated in diffuse large B-cell lymphomas. Of all, *FBXW7* was the only gene recurrently mutated and all mutations were found in highly evolutionarily conserved regions and considered damaging by PolyPhen and MutationTaster (42). This data supports the notion that ‘CLL-associated’ gene mutations are not necessarily clinically relevant when detected in MBL.

Along these lines, in our recent characterization of the genetic landscape in both subtypes of MBL as well as indolent CLL, we were able to detect gene mutations in CLL putative driver genes in all 3 entities, yet these were infrequent and did not have any obvious impact on disease progression after a prolonged follow up. On these grounds, we proposed that gene mutations may represent late events related mostly to disease progression, whereas interactions between the cell clone and its microenvironment, such as those mediated through the B cell receptor (BcR), could represent the major driver in the early stages of the natural history of CLL (40).

## B cell clonal dynamics in MBL: monoclonal versus multiclonal cases

Findings from low-throughput studies using mostly subcloning techniques studies reported that oligoclonality is a common feature of ‘CLL-like’ MBL (43), present in both LC-MBL and HC-MBL cases. In 2011, similar findings were obtained in the first relevant NGS study of 9 individuals with HC-MBL in whom multiple, immunogenetically independent MBL clones were identified, clearly suggesting distinct origins (44). The coexistence of multiple B cell expansions was also observed in a subsequent study as well, where the authors concluded that different subsets of normal B cells in the same individual may be targets for oncogenetic events involved in lymphomagenesis, arguing that bi- and multi-clonal MBL cases may be associated with distinct chronic antigen-driven immune responses (44),. This could be particularly frequent at earlier stages (LC-MBL), further evidence for the potential reactive nature of MBL among individuals with normal lymphocyte counts, prior to the stepwise acquisition of genomic alterations and the (infrequent) progression to HC-MBL and CLL (45).

Of note, in the latter study, the median numbers of clonal B cells in both ‘CLL-like’ HC-MBL and CLL were significantly lower in multiclonal than in monoclonal cases (41). In contrast, the median number of clonal cells in ‘CLL-like’ LC-MBL was significantly higher in multiclonal than monoclonal cases. Yet, longitudinal analysis showed a significant increase in size in monoclonal compared to bi- and multi-clonal LC-MBL cases (86% versus 54%, respectively; p=0.004). In most studies, no statistically significant differences in IGHV gene usage were identified in monoclonal versus multiclonal cases. Interestingly, the number of multiclonal cases with stereotyped BcR IG was significantly higher than that of monoclonal cases. Finally, the frequency of cytogenetic alterations as well as genetic complexity in both ‘CLL-like’ MBL and CLL clones from multiclonal cases was significantly lower than that of monoclonal cases from these entities (45).

**Figure 3:**
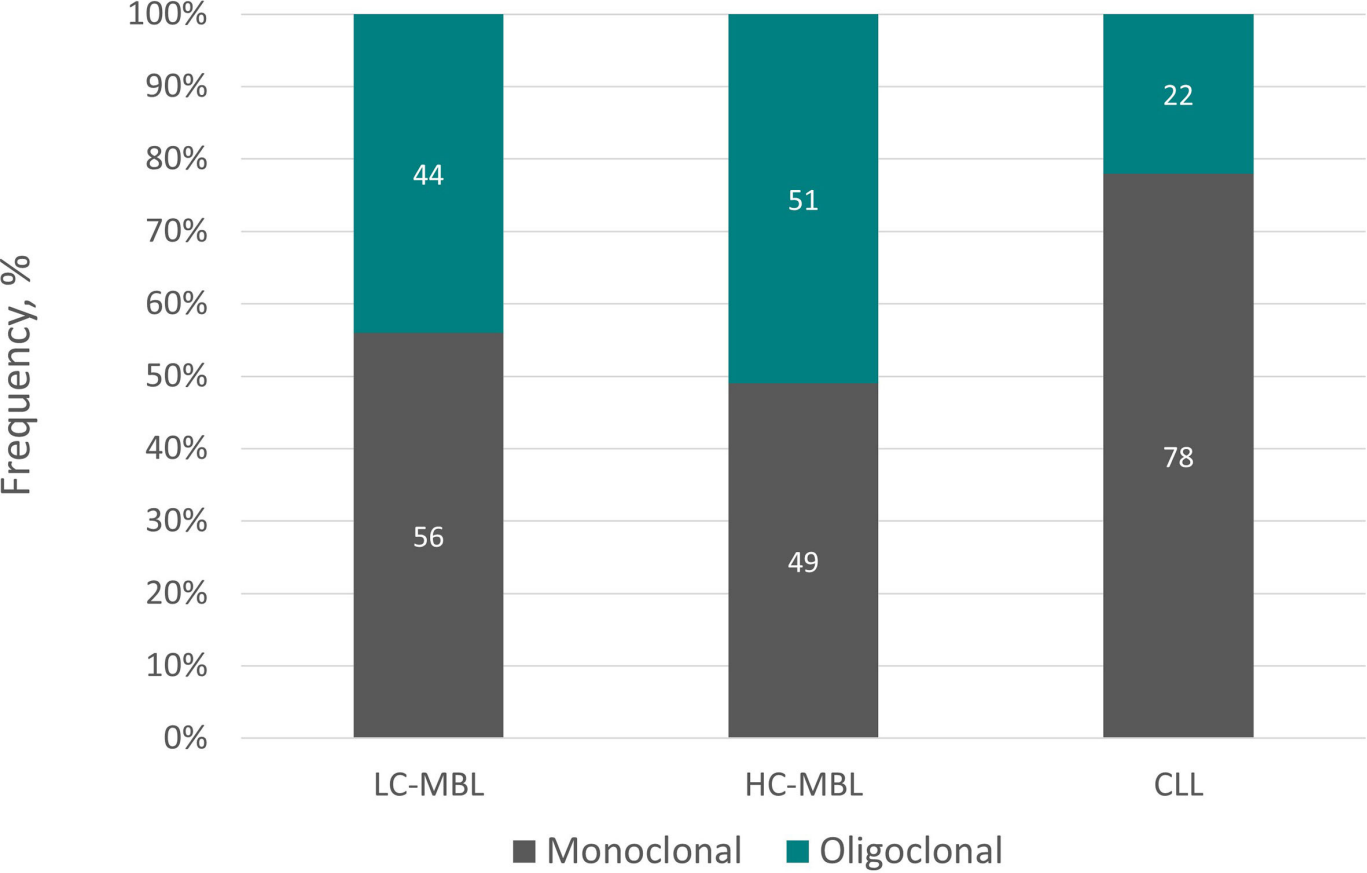
Monoclonal versus oligoclonal case distribution in LC-MBL, HC-MBL and CLL. Data regarding LC-MBL were extracted from Agathangelidis et al. (46) and Henriques et al. (26), whereas the data concerning HC-MBL and CLL were obtained from the latter.

More recently, employing NGS, we systematically assessed the immunogenetic characteristics of LC-MBL. We found that 75% samples displayed a monoclonal profile characterized by the presence of a single clonotype dominating the repertoire (frequency range: 42.5-97.9%), whereas the remaining 25% of samples turned out to be oligoclonal (46). Clonality levels seemed to correlate with clone size, in line with previous studies reporting a lower frequency of oligoclonality along the transition from LC-MBL to HC-MBL and, eventually, CLL (47). **Figure 3** summarizes graphically the distribution of monoclonal and oligoclonal cases among the 2 subtypes of MBL and CLL.

Arguably, differences in clonality patterns among LC-MBL cases could indicate that these are captured at different stages along the ontogenetic trajectory. In this scenario, LC-MBL could initially involve a polyclonal B cell population that at some point acquired the CLL phenotype due to persistent antigenic stimulation. Microenvironmental interactions would induce further proliferation, eventually favoring the acquisition of genomic lesions, hence underlying the progressive transition to oligoclonal and, eventually, monoclonal LC-MBL cell populations. This evolutionary path could go in parallel with a continuous increase in clonal size, perhaps explaining the existence of multiclonal HC-MBL and CLL cases (25, 48).

## LC-MBL has a different immunogenetic profile from either HC-MBL or CLL

The first studies reporting significant IGHV gene usage biases in the repertoire of CLL date from the 1990s (49). Subsequent studies in larger patient cohorts (50–52) cemented the notion that skewing of the IGHV repertoire in CLL results from strong selective pressures acting on the CLL progenitor cells by of a restricted group of antigens.

In order to obtain more insight into the reasons/ontogenetic timing repertoire skewing, several groups undertook comparative analyses of the IGHV gene repertoire in CLL and MBL. Early studies compared Rai 0 CLL with HC-MBL, yet the differences found were not statistically significant (17, 21). However, when comparisons extended to LC-MBL, that latter was found to display a distinct profile, including frequent expression of the IGHV4-59 and IGHV4-61 genes (rather infrequent in CLL), as well as pronounced scarcity of the IGHV4-34 and IGHV1-69 genes (26, 53). These findings suggested that HC-MBL and indolent CLL are closely related at the immunogenetic level, whereas LC-MBL is characterized by a rather unique repertoire, raising questions regarding its precise relationship to CLL (17, 43).

**Figure 4:**
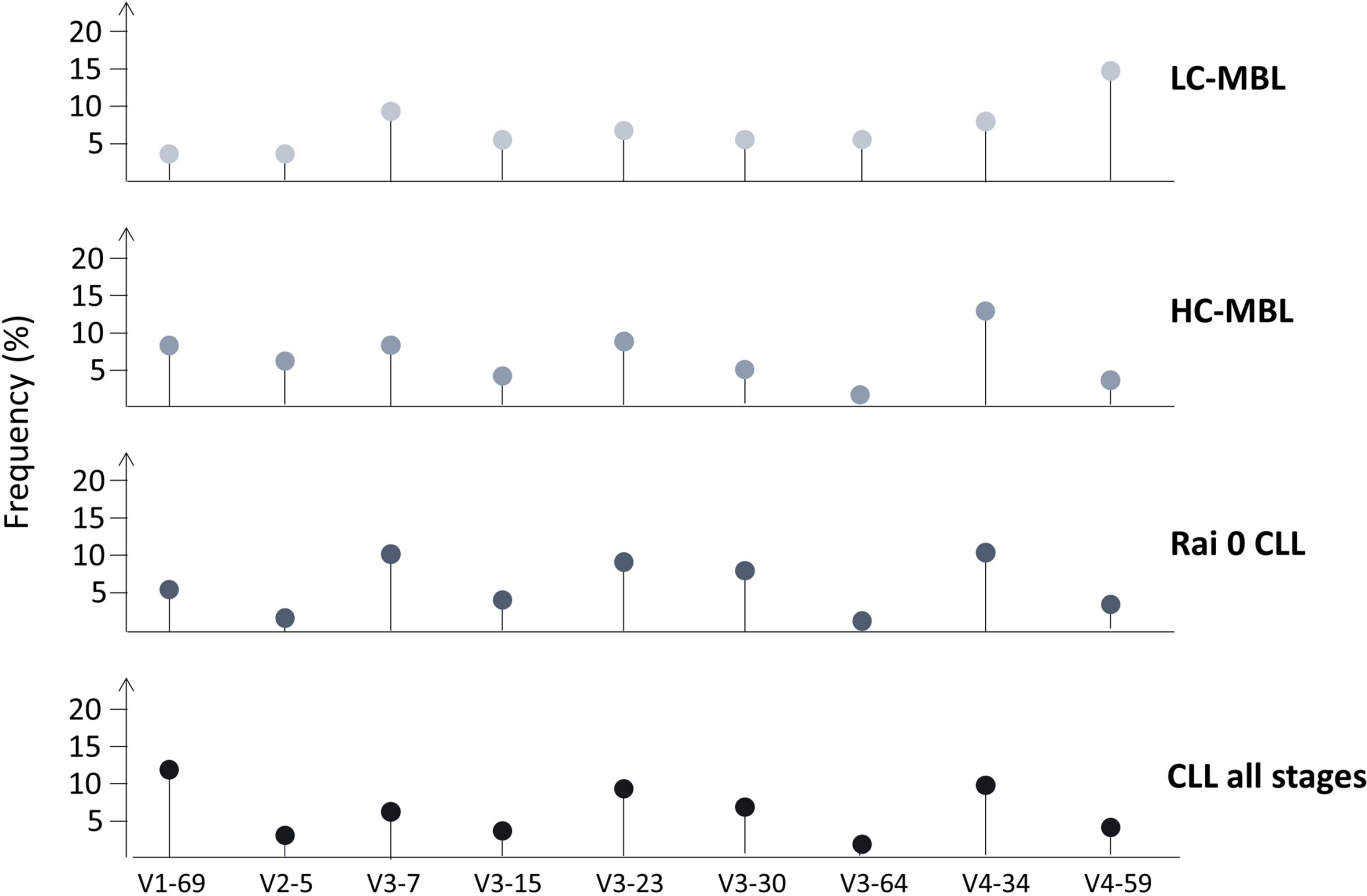
IGHV gene repertoire differences between LC-MBL, HC-MBL, Rai 0 CLL as well as in CLL from all stages. Data was obtained from Vardi et al. (54).

In the largest relevant study, our group applied Sanger sequencing in a cohort of 333 ‘CLL-like’ MBL cases and reported significant differences in the IGHV gene repertoire between HC-MBL versus LC-MBL; several genes were frequent only in the former (namely IGHV1-69, IGHV2-5, IGHV3-23, IGHV3-33, IGHV3-48 and IGHV4-34), whereas the IGHV4-59/61 genes was significantly more frequent in the latter. Comparison of both MBL subtypes against exclusively Rai-0 CLL cases or CLL cases from all stages disclosed several significant differences, again highlighting the distinct nature of the LC-MBL repertoire (**Figure 4**) (54). These findings strengthened the hypothesis that LC-MBL may not constitute a true pre-leukemic state but rather represent a manifestation of immune senescence.

More recently, we re-appraised the immunogenetic profile of LC-MBL employing a high-throughput approach (46). More particularly, employing NGS, we studied the IGHV gene repertoire from clonal and normal B cell samples from 23 individuals with LC-MBL, which we compared against the corresponding repertoires of naïve and memory B cell samples from 6 healthy individuals. The most intriguing finding of our study concerned the stronger repertoire skewing in normal B cells from individuals with LC-MBL compared to healthy individuals. This argues for different selection processes and functions of these B cell subpopulations in LC-MBL compared to healthy individuals (55).

## Different patterns of somatic hypermutation in MBL compared to CLL

The SHM status of the clonotypic IGHV gene is now considered one of the most accurate biomarkers for clinical decision making in CLL (30). Based on the SHM status, CLL patients can be robustly classified CLL into 2 distinct groups: (i) CLL with unmutated IGHV genes (U-CLL), with few or no SHM [i.e. cases with a germline identity equal to or higher than 98%]; and, (ii) CLL with mutated IGHV genes (M-CLL), with a considerable SHM load and a germline identity below 98%. This classification scheme holds strong prognostic relevance, with U-CLL cases generally experiencing a worse prognosis and a more aggressive disease accompanied by early need for treatment compared to M-CLL (56, 57). It has been postulated that these two groups might also have distinct ontogeny, with M-CLL possibly deriving from B cells that have been activated by antigen(s) and have participated in the germinal center (GC) reaction, and U-CLL cases possibly originating from B cells following GC-independent maturation pathways (48, 58).

**Figure 5:**
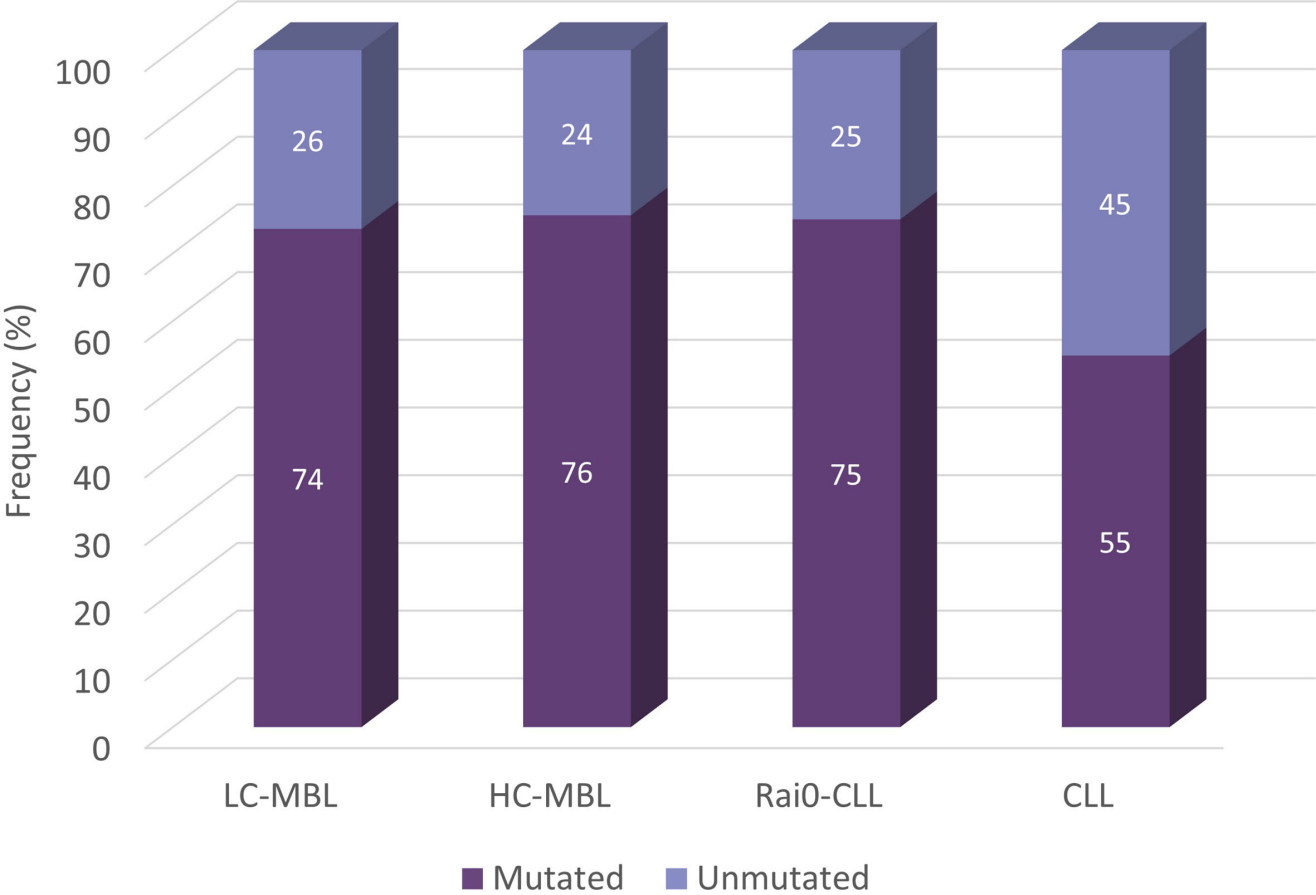
Somatic hypermutation status in cohorts from LC-MBL, HC-MBL and CLL. Data was obtained from Vardi et al. (54).

Following the same, CLL-relevant 98% identity cut-off value, several studies analysed the distribution of mutated and unmutated cases in MBL. In our series, out of the 355 IGH gene rearrangements analysed from 333 ‘CLL-like’ MBL cases, 267 (75.2%) were classified as mutated, while the rest (24.8%) were assigned to the unmutated subgroup. The distribution of unmutated and mutated IGH gene rearrangements in LC-MBL and HC-MBL was similar, and also comparable to Rai 0-CLL (26.4%, 24.4% and 25%, respectively). These 3 groups, however, differed significantly when compared against a CLL cohort containing 7,424 CLL patients from all stages, in which the frequency of unmutated cases was 45.1% (**Figure 5**) (54). In line with the above, both HC-MBL and Rai 0-CLL unmutated cases have been found to display distinct gene and miRNA expression profiles from the mutated MBL/CLL cases, independently of their classification (21).

## Stereotyped BcR IG are infrequent in LC-MBL

A distinctive feature of the IG gene repertoire of CLL concerns the presence of (quasi)identical BcR IG shared by different patients, a phenomenon termed BcR IG stereotypy. At odds with serendipity, whereby stereotyped BcR IG would be found with a negligible frequency (in the range of 10^-12^-10^-16^), stereotypy occurs in CLL at a remarkable frequency of 41% (59). Groups of patients expressing such restricted BCR IG are known as stereotyped subsets: they can be found in both U-CLL and M-CLL and vary in size from a handful to hundreds of cases, in which case they are deemed ‘major’ (52, 59). Notably, similarities between cases in stereotyped subsets extend from the primary IG sequences to biological and clinical profiles. Indeed, consistent clinical outcomes have been reported for patients assigned to the same subset (60–62), likely linked to consistent biological profiles: these pertain, amongst others, to distinctive gene expression, cytogenetic aberrations, BcR structure and signalling capacity, epigenetic modifications and antigenic reactivity profiles (63–71) that are also distinct between subsets but also from non-subset cases even of the same mutational category (i.e. U-CLL or M-CLL).

**Figure 6:**
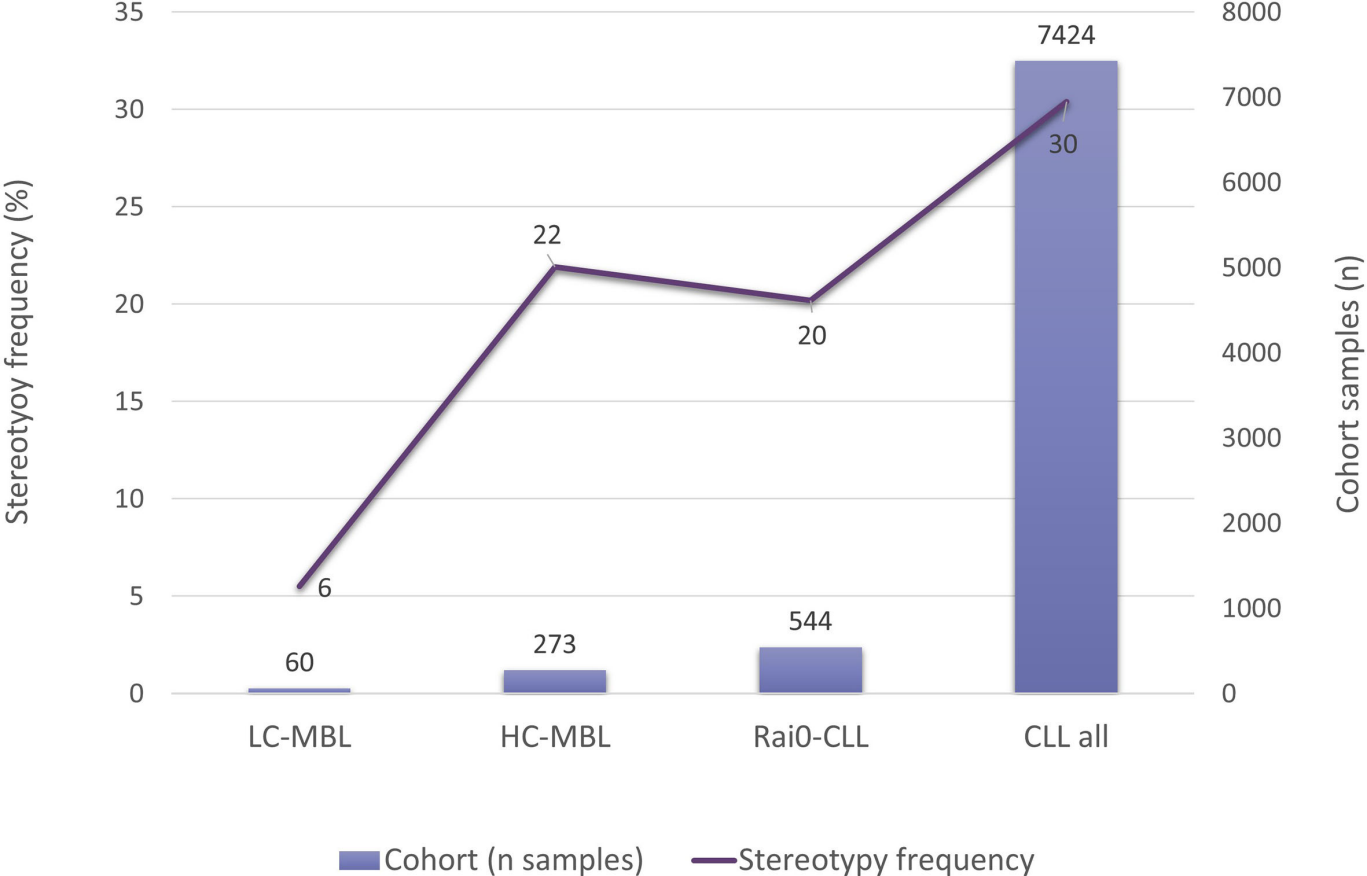
Frequency of BcR IG stereotypy in LC-MBL, HC-MBL, Rai 0 CLL and CLL from all clinical stages. Data was extracted from Vardi et al. (54).

In this context, investigating whether ‘CLL-like’ stereotyped BcR IG could be detected also in MBL was a logical next step. When comparing HC-MBL with Rai 0-CLL, no significant differences were found, whereas, in sharp contrast, stereotyped BcR IG were exceedingly scarce in LC-MBL (17, 21). Interestingly, a correlation between the incidence of stereotypy and the absolute count of ‘CLL-like’ cells was identified: 5.5% in LC-MBL, 21.9% in HC-MBL, 20.2% in Rai 0-CLL, and 30.4% in CLL of all clinical stages (**Figure 6**) (54).

The scarcity of BcR IG stereotypes in LC-MBL was also evident in our recent high-throughput study. Of the few stereotyped clonotypes, virtually all were classified to minor subsets with a small minority corresponding to stereotypes defining major CLL stereotyped subsets, particularly those associated with aggressive disease. On these grounds, we proposed that LC-MBL is immunogenetically distinct from CLL, at least for those cases with aggressive clinical behavior (46).

## T cells in MBL and CLL

Multifarious abnormalities in the T cell compartment constitute a well characterized feature of the tumor microenvironment in CLL (72–75). However, although our understanding of the interactions between T cells and CLL leukemic cells is continuously growing, several important questions remain, not lest regarding the precise role of T cells i.e. whether they exert pro- or anti-neoplastic actions.

CLL is characterized by a relative loss of naïve CD4^+^ T cells accompanied by enrichment of antigen-experienced, memory and effector CD4^+^ T cells (73, 76). CD4^+^ T cells in CLL express higher levels of PD-1, human leukocyte antigen (HLA)-DR and Ki67, which are all activation markers (73, 77, 78). *In vitro* as well as *in vivo* studies in xenograft mouse models suggested that CD4^+^ T cells could enhance the survival and proliferation of clonal cells in CLL, a finding that was supported by correlations between CD4^+^ T cell counts and clinical outcome (79, 80).

CD8^+^ T cells from patients with CLL display high expression of PD-1 and other inhibitory receptors, accompanied by defective immune synapse formation (also occurring in CD4^+^ T cells as well) (78, 81). For these reasons, CD8^+^ T cells in CLL have been described as “pseudoexhausted” (78). Relevant experiments in mice showed that the exhaustion of T cells was anatomically restricted, being observed mainly in spleen samples and to a much lower extent in the blood (81). Impaired immune synapse formation was observed *ex vivo* when T cells from healthy donors were co-cultured with clonal CLL cells, suggesting that this defect was largely induced by the CLL cells themselves. Of interest, blocking inhibitory ligands, such as CD200, PD-L1, or CD276 with neutralizing antibodies led to restored immune synapse formation capacity (82). Regarding the biological function of CD8^+^ T cells in CLL, apparently they may recognize tumor specific antigens, however they ultimately fail to control the disease likely due to their functional exhaustion (76).

Characteristic T cell receptor (TR) gene repertoire restrictions have been established in CLL, mainly in the CD8^+^ but also in the CD4^+^ compartment (83). Even though clonal expansions occur frequently in the T cell repertoire of the elderly, restrictions were more pronounced in CLL, indicating the existence of stronger and perhaps different selective pressures. Whether these pressures are exerted by the same antigens interacting with the CLL clone or by CLL-derived antigens remains to be elucidated (83, 84).

Comparative analysis between HC-MBL, U-CLL and M-CLL cases revealed similar CD4^+^ T cell counts in all three conditions. In contrast, the CD8^+^ T cell count was higher in U-CLL compared to M-CLL patients and significantly higher than that of HC-MBL (85). Interestingly, both CD4^+^ and CD8^+^ clonal T cell expansions were identified in HC-MBL, similar to CLL. CD4^+^ T cell clonal expansions appeared to follow the numerical increase of clonal B cells, thus being more pronounced in CLL compared to HC-MBL (85). This finding suggests that CD4^+^ TR repertoire restrictions may be somehow influenced by the size of B cell clonal expansions, occurring early in clonal evolution and increasing concurrently with tumor progression. The fact that CLL displays higher T cell repertoire restrictions could also reflect the loss of effector T cell clones restraining CLL clonal expansions. In line with this, treatment with the Bruton’s tyrosine kinase inhibitor ibrutinib, led to an increase in the T cell repertoire diversity; this suggests that the eradication of the CLL malignant cells sets the stage for T cell reconstitution (86).

Concerning the clonality of the CD8^+^ T cell fraction, no significant differences were detected between HC-MBL and CLL (87). Furthermore, a longitudinal analysis showed that T cell clones, mainly from the CD8^+^ compartment, persisted over time in all MBL samples analyzed, similar to what was previously reported in CLL (83, 88), suggesting that interactions between CLL and T cells are evident prior to/at the early stages of CLL development (87). Moreover, decreased numbers of CD4^+^CD8^+^ double positive T cells were identified in HC-MBL compared to healthy control samples, alluding to impaired immunosurveillance which may actually favor the emergence of MBL clones. Furthermore, Tregs appeared increased in CLL, but not in MBL, compared to healthy controls (89).

Regarding LC-MBL, early studies reported an increase in the size of clonal T cell populations compared to the general population, especially for double positive CD4^+^CD8^+^ T cells, suggesting a general deregulation of the immune system, possibly related to chronic antigenic stimulation (90). Recently, a follow-up study of LC-MBL revealed statistically significant increase of total T cells as well as CD4^+^, CD8^+^ and the double negative cell subpopulations over time. Interestingly, LC-MBL with larger clone sizes over time also showed significantly higher (P<0.05) numbers of the distinct normal residual T cell subsets at follow up compared to the baseline, especially for CD4^+^ T cells (79, 91). This might imply that signals emanating from immune cells of the CLL microenvironment, potentially promoting activation, proliferation and/or survival of B cells, could contribute to the expansion of ‘CLL-like’ B cell clones at the earliest stages of CLL (25).

In our recent study of the TR beta chain gene repertoire in LC-MBL by NGS, we reported more pronounced T cell expansions in LC-MBL compared to aged-matched healthy individuals, yet lower than CLL. We attributed this finding to different antigenic pressures, leading to more pronounced T cell expansions in CLL versus LC-MBL versus healthy individuals (92). Moreover, a significant correlation between the level of T cell clonality and the size of the MBL clone was shown, in line with previous findings in CLL, further supporting the notion that T cell expansions could be driven by CLL-associated antigens (83, 88). Pairwise comparisons between all entities revealed distinct TRBV gene repertoire biases; this could perhaps imply that different antigen selection pressures operate in LC-MBL, CLL or healthy individuals. The latter assumption was further supported by the fact that shared clonotypes between different entities were scant. The nature of the implicated antigens and whether they are related to the expanded ‘CLL-like’ cell clones remains to be clarified (92).

## The numbers of residual normal B cells are reduced prior to CLL onset

Several defects in both the innate and the adaptive immune responses have been identified in CLL, including hypogammaglobulinemia (93). The exact mechanisms underlying these defects are poorly characterized, whereas even less information is available regarding the disease stage during which they take place. In this context, Criado and colleagues (55) studied the residual normal B cell compartment in the blood of individuals with LC-MBL and HC-MBL compared to Rai 0-CLL in order to get insight into the mechanisms that may contribute to the emergence of hypogammaglobulinemia in CLL. Both HC-MBL and Rai 0-CLL showed significantly reduced normal B cell counts, mostly at the expense of pre-GC B cells, both immature and naïve, suggesting diminished production and/or release of these cells in the circulation at the early stages of CLL. Considering that no such decrease has been identified in circulating immature and naïve B cell counts in relation to ageing (94), the decreased numbers of circulating pre-GC B cells in individuals with HC-MBL alludes to impaired production of newly generated B cells in the BM, even prior to the development of CLL (55).

When focusing on the memory B cells (MBC) and plasma cells (PC), there were no significant differences in either MBL (both subtypes) or CLL compared to healthy controls with the exception of PC counts between HC-MBL and the healthy controls. Of note, both MBC and PC showed significantly different distribution among B cell subsets, expressing distinct IG subclasses in both MBL and CLL. Of importance, the extent of these changes was greater from LC-MBL to HC-MBL and finally Rai0-CLL. Thus, the possibility of a progressive impairment of B cell responses driven by newly encountered antigens along the transition from LC-MBL to HC-MBL and finally Rai 0-CLL was proposed; impaired pre-GC B cell production has been considered as largely responsible, since it could lead to a progressive reduction of the diversity of the BcR IG repertoire from LC-MBL to HC-MBL and Rai 0-CLL (55). This would be in line with other features of CLL but also MBL, such as the launch of active, but silent, responses against common pathogens, particularly viruses such as cytomegalovirus (CMV) and the Epstein Barr virus (EBV), as well as bacteria such as *S. pneumoniae* (95). Additional, longitudinal studies in larger MBL and CLL cohorts would be necessary to support this hypothesis.

## Conclusions

MBL detection is based on the identification of B cell expansions in the blood circulation with the characteristic “CLL-specific” phenotype, yet of a smaller size than the one required for CLL diagnosis. This criterion, based solely on a mathematical cutoff is devoid of any biological context, perhaps hampering our understanding of the mechanisms driving disease onset. Thus, the in-depth characterization of MBL holds great potential for understanding the mechanisms that represent major drivers in the process of its transformation to CLL.

Nowadays, it is well established that CLL is characterized by a highly complicated genomic landscape, including a series of cytogenetic aberrations as well as gene mutations. That said, the overall simple genetic background of MBL pointed towards the BcR IG as one of the major “players” prior to disease onset. The study of BcR IG clonality, especially through NGS-based methodologies, revealed much a much higher frequency of oligoclonality in MBL, especially LC-MBL, compared to CLL, most likely reflecting different staged along the ontogenetic trajectory. Relevant to mention, the BcR IG repertoire in HC-MBL was similar to CLL regarding both the IGHV gene repertoire as well as BcR IG stereotypy, whereas LC-MBL showed a highly distinct repertoire characterized by the dominance of different IGHV genes and a very low prevalence of stereotypes, alluding to different antigenic pressures shaping the BcR IG repertoire in LC-MBL versus HC-MBL and CLL. Further support for an early role of the microenvironment in CLL ontogenesis came from the study of the TR repertoire in both LC- and HC-MBL. Similar to CLL, T cell expansions were evident in both subtypes of MBL yet at a lower extent. As above, the TR repertoire was characterized by different biases in the repertoire of TRBV genes and the absence of shared clonotyped among MBL and CLL pointing towards different selection mechanisms. Last, the study of residual B cells in MBL and CLL revealed an impaired production of newly generated B cells in the BM, even prior to the development of CLL, as well as a progressive impairment of B cell responses driven by newly encountered antigens along the transition from LC-MBL to HC-MBL and finally Rai 0-CLL that could account for the clinical impact of MBL detection.

Overall, even if major questions still remain, parallel analysis of the B cell clone(s) and the tumor microenvironment in MBL holds strong potential for characterizing the mechanisms that are required for the emergence and malignant transformation of a monoclonal B cell population.

## Author Contributions

All authors contributed to the article and approved the submitted version. CG, LZ-I, and AI wrote the manuscript. AC, KS, and AA edited the text and gave final approval.

## Funding

This work was supported in part by the Hellenic Foundation for Research and Innovation (HFRI) and the General Secretariat for Research and Technology (GSRT), under grant agreement No 336 (Project CLLon); COSMIC, a Marie Curie European Training Network funded from the European Union’s Horizon 2020 research and innovation programme under grant agreement no. 765158; the project ODYSSEAS (Intelligent and Automated Systems for enabling the Design, Simulation and Development of Integrated Processes and Products) implemented under the “Action for the Strategic Development on the Research and Technological Sector”, funded by the Operational Programme “Competitiveness, Entrepreneurship and Innovation” (NSRF 2014-2020) and co-financed by Greece and the European Union, with grant agreement no: MIS 5002462; NEoteRIC, funded from the European Union’s Horizon 2020 research and innovation programme under grant agreement No 871330; the Greek Precision Medicine Network (GPMN), a mission of the Research and Innovation sector of the Ministry of Education, Research and Religious Affairs of Greece.

## Conflict of Interest

The authors declare that the research was conducted in the absence of any commercial or financial relationships that could be construed as a potential conflict of interest.

## Publisher’s Note

All claims expressed in this article are solely those of the authors and do not necessarily represent those of their affiliated organizations, or those of the publisher, the editors and the reviewers. Any product that may be evaluated in this article, or claim that may be made by its manufacturer, is not guaranteed or endorsed by the publisher.
